# Screen-Printed Electrodes Modified with Metal Phthalocyanines: Characterization and Electrocatalysis in Chlorinated Media

**DOI:** 10.3390/s20133702

**Published:** 2020-07-02

**Authors:** Daniel Antuña-Jiménez, David Ibáñez, María Begoña González-García, David Hernández-Santos, Pablo Fanjul-Bolado

**Affiliations:** Metrohm DropSens S.L., Vivero Ciencias de la Salud, C/Colegio Santo Domingo de Guzmán s/n, 33010 Oviedo (Asturias), Spain; daniel.antuna@metrohm.com (D.A.-J.); david.ibanez@metrohm.com (D.I.); begona.gonzalez@metrohm.com (M.B.G.-G.); david.hernandez@metrohm.com (D.H.-S.)

**Keywords:** metal phthalocyanines, screen-printed electrodes, electrocatalysis, electrochemical detection, voltammetric sensors, Raman spectroscopy, chlorine detection

## Abstract

Metal phthalocyanines are well-known sensing phases with applications in different scientific fields due to their interesting properties. Detailed characterization by Raman spectroscopy was performed in order to study the shifting of the vibrational bands related to the coordination sphere of each metal phthalocyanine. In this work, a study involving the use of screen-printed electrodes (SPEs) with various metal phthalocyanines to electrochemically detect and quantify chlorine (Cl_2_) gas is presented. The Cl_2_ gas was generated in-situ via oxidation of the chloride present in form of aqueous salt solutions. The developed method offers not only the possibility to quantify chlorine, but also to discriminate among several chlorinated species due to the changes observed in the voltammetric profiles associated with the interaction between the specie assayed and the phthalocyanine metallic center. Optimization of detecting parameters was also performed to apply this procedure for the quantification of chlorine generated from commercial chlorine tablets. The development of this proof of concept shows interesting possibilities and easy-to-use applications with novel on metal phthalocyanines based SPE sensors.

## 1. Introduction

Phthalocyanines are planar aromatic systems analogous to porphyrins but with an important feature, the central cavity of the ring can be replaced with a metal ion bonded to the four pyrrole nitrogen atoms. About 70 different metals [1] can be used as the central ion, opening new gates in the generation of phthalocyanines with tunable properties. Their excellent thermal and chemical stability as well as their outstanding optical properties turn them into very promising materials for a huge variety of applications such as preparation of dyes and pigments [2], optical devices [3], solar cells [4], gas sensors [5], optical data storage [6], electrochromic devices [7], electrocatalytic applications [8,9] and biosensors [10].

As it is well known, Raman spectroscopy is one of the most interesting techniques for characterizing different materials, compounds and electrochemical processes [11,12,13,14,15,16]. Indeed, it is a potential approach for characterizing metal phthalocyanines because it provides structural information related to their molecular vibrational modes [17]. The resonance Raman effect of metal phthalocyanines allows obtaining well-defined bands in Raman spectra, being particularly interesting for their ability of identification in pigments, solutions and biological matrices [18] and the study of their structural modifications [19]. Although the position of most vibrational Raman bands does not depend on the metal ion, the spectral region between 1350 and 1550 cm^−1^ is considered as a fingerprint since it is very sensitive to the central ion, particularly in unsubstituted metal phthalocyanines [17,20]. Furthermore, the position of Raman bands in the fingerprint region not only depends on the metal ion, but also on its solvation sphere. Taking into account that Raman bands are assigned to specific vibrational modes, the hydration of the metal ion affects the position of these bands. Since Raman spectroscopy is a fingerprint technique, it has been used in this work as a quick tool to obtain a detailed characterization of dry and wet metal phthalocyanines as well as to study if the change of the solvation sphere could affect the position of Raman bands.

Among the rich bibliography related to gases and metal phthalocyanines [21,22], detection of chlorine gas with copper phthalocyanines was previously explored in electrochemical [23] or optical devices [24]. These studies were usually developed under a gas flow employing high temperature conditions [25], although temperature can be decreased to work at more friendly room conditions, depending on the film assayed [26]. In this way, the main aim of this work is to develop an easy generation of chlorine in solution followed by its subsequent detection with several metal phthalocyanines as sensing phases. As proof of concept, several chlorinated species were assayed in order to explain their electrocatalytic behavior onto the metal phthalocyanine modified electrodes. The methodology developed in this work involves electrochemical generation of chlorine in a drop of solution by oxidation of a chlorinated reagent. Diluted gas is finally detected by following its reduction process onto the sensing surface. Electrogeneration parameters were optimized in order to improve detection of chlorine with the subsequent voltammetric reduction. This protocol was tested using commercial chlorine tablets to provide new applications of metal phthalocyanines sensing phases in real samples.

## 2. Materials and Methods

### 2.1. Reagents

Perchloric acid (70–72%), hydrochloric acid (25%) and chlorine tablets (Ref. ZWCL01F50) were purchased from Merck (Madrid, Spain). Sodium hydroxide and potassium chloride were purchased from Sigma-Aldrich (Madrid, Spain). All chemicals were analytical grade and used as received. Aqueous solutions were prepared using ultrapure water (Direct-Q™ 5 system, Millipore, Madrid, Spain).

### 2.2. Instrumentation

Screen-printed Co-Phthalocyanine (DRP-410), Cu-Phthalocyanine (DRP-110CUPH), Fe-Phthalocyanine (DRP-110FEPH) and Mn-Phthalocyanine (DRP-110MNPH) electrodes from Metrohm DropSens (http://www.metrohm-dropsens.com/, Oviedo, Asturias, Spain) were used to perform the electrochemical measurements. The electrodic systems consist of a flat ceramic card with a circular carbon working electrode (4 mm diameter with a 0.13 cm^2^ surface area) modified with metal phthalocyanines, a carbon auxiliary electrode and a silver pseudo-reference electrode. DRP-110 electrodes were used in blank experiments to show the behavior of unmodified carbon substrate. Electrodes employed in this work are single use due to the methodology developed that will be explained in subsequent sections.

Electrochemical measurements were performed at room temperature using a multi potentiostat/galvanostat µStat 8000 (Metrohm DropSens) controlled by DropView 8400 software.

Raman measurements were carried out with SPELEC RAMAN (Metrohm DropSens), a compact instrument with a laser source of 785 nm. This instrument was connected to a bifurcated reflection probe (DRP-RAMANPROBE, Metrohm DropSens) and a specific cell for screen-printed electrodes SPEs (DRP-RAMANCELL, Metrohm DropSens) was used. The SPELEC RAMAN instrument was controlled by DropView SPELEC software.

### 2.3. Methods

All Raman spectra were recorded at room temperature using 10 s as the integration time, laser power of 185 mW and spot size of 190 µm diameter. Spectra were corrected by baseline subtraction.

The methodology developed for chlorine sensing was carried out in two electrochemical steps. In the first step, chlorine gas was electrogenerated “in situ” with the application during a fixed time of a high potential capable to oxidize chloride, present in the solution, to chlorine. Under optimal conditions, a 50 µL solution drop was placed onto the metal phthalocyanine based SPE and an initial potential of +1.20 V was applied for 3 s. In the second step, the detection of electrochemically generated chlorine was achieved with a linear sweep voltammetry (LSV) scanning from +1.20 to +0.25 V in order to reduce the chlorine diluted in solution. For optimization purposes, several parameters were evaluated as shown in following sections.

Additionally, coulometric experiments were carried out to estimate the concentration of chlorine electrogenerated onto the electrodic surfaces. In each optimization step, charge was measured when different initial potentials and different times of electrogeneration were applied. The concentration of chlorine was calculated from charge data applying Faraday’s law and assuming 100% of total oxidation efficiency from chloride to chlorine. The charge values were also obtained with a carbon naked electrode (DRP-110) for each experimental condition assayed in order to subtract the charge generated with metal phthalocyanine modified carbon electrodes. The concentrations of chlorine obtained in each experiment are shown in each plot where the optimization of parameters detection is discussed. In the case of commercial tablets, no coulometric assays are carried out.

## 3. Results and Discussion

### 3.1. Metal Phthalocyanines Based SPEs Characterization by Raman Spectroscopy

**Figure 1:**
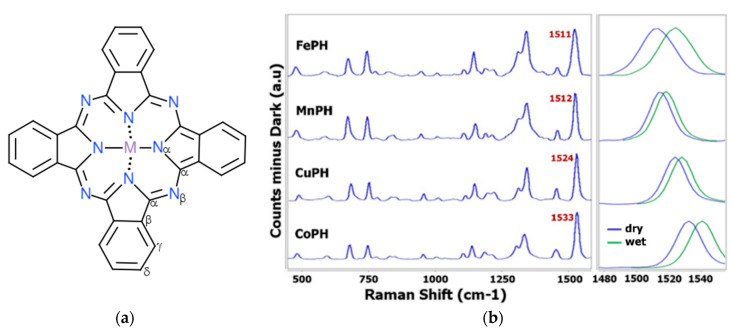
(**a**) Chemical structure of metal phthalocyanines. M = Co, Cu, Mn and Fe. (**b**) Raman spectra of dry (blue line) and wet (green line) CoPH (DRP-410), CuPH (DRP-110CUPH), MnPH (DRP-110MNPH) and FePH (DRP-110FEPH). Spectra were recorded using 10 s as the integration time and corrected by baseline subtraction.

Raman spectra of dry SPEs modified with metal phthalocyanines (Figure 1a) were obtained focusing the laser spot on the electrode surface according to the focal distance of the Raman probe. Figure 1b (blue line) shows that the characteristic spectra of dry commercial SPEs modified with CoPH, CuPH, MnPH and FePH are close, similar because the phthalocyanines share the same chemical structure and only the metal central ion is different in each one of them.

Comparison of Raman spectra obtained in the characterization of dry DRP-410, DRP-110CUPH, DRP-110MNPH and DRP-110FEPH is shown in Table 1. As can be noticed, the position of the major part of the vibrational bands does not depend on the metal ion and only a slight shifting is observed between them. However, the region between 1350–1550 cm^−1^ is metal dependent and the position of Raman bands in this spectral range is defined by the central ion [17,20]. In that way, Table 1 and Figure 1b show that an interesting band to identify different metal phthalocyanines is the band that is located between 1510 and 1540 cm^−1^ because the characteristic band of CoPH is centered at 1533 cm^−1^, CuPH at 1524 cm^−1^, MnPH at 1512 cm^−1^ and FePH at 1511 cm^−1^. This band is assigned to the displacements of C-N-C bridge bonds of the phthalocyanine macrocycle and is sensitive to the ion due to a change in shape of the entire ring [17,27]. Although the characteristic bands of FePH and MnPH are located very close, the shape is different for each of them. Figure 1b shows that Raman band associated with FePH at 1511 cm^−1^ is wider than vibrational band of MnPH at 1512 cm^−1^. Then, not only the position but also the shape of Raman bands allows us to make the identification of metal phthalocyanines.

In addition, Raman characterization of wet electrodes was also performed. Different media, such as ultrapure H_2_O, 0.1M KCl, 0.1 M HCl, 0.5 M HClO_4_ and 0.1 M NaOH aqueous solutions were used. Spectroscopic information shows that the same up-shiftings of the characteristic Raman bands are observed: CoPH shifts from 1533 to 1541 cm^−1^, CuPH from 1524 to 1528 cm^−1^, MnPH from 1512 to 1518 cm^−1^ and FePH from 1511 to 1523 cm^−1^ (green line in Figure 1b).

Hence, the shifting of these bands does not depend on the selected media, but the metal ion is a key factor of this effect. Taking into account that Raman bands are related to vibrational modes, the observed shiftings are directly related to their solvation modification due to the introduction of solvent molecules in the structure. Indeed, the insertion of substituents can create steric crowding, that is released by the nonplanar deformation of the macrocycle [29]. Therefore, the Raman band related to the central ion is a good marker for changes in the metal ion environment. Raman measurements were also performed when the electrodes previously characterized with solution were completely dry again. Under these experimental conditions, Raman shift recovers the initial value (1533 cm^−1^ for CoPH, 1524 cm^−1^ for CuPH, 1512 cm^−1^ for MnPH and 1511 cm^−1^ for FePH); then Raman shifting due to the modification of the solvation sphere is a reversible process.

### 3.2. Electrocatalytic Application with Chlorinated Species

**Figure 2:**
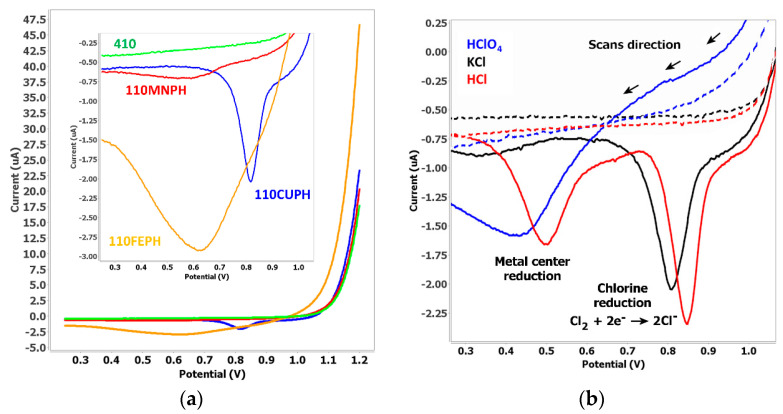
(**a**) Electrochemical behavior of screen-printed electrodes (SPEs) modified with different metal phthalocyanines in 50 mM KCl solution. (**b**) linear sweep voltammetry (LSV) of 100 mM chlorinated species assayed with DRP-110CUPH. Dotted lines correspond to the DRP-110 electrodes response.

Various metal phthalocyanines were tested as sensing phases in KCl by means of an electrogeneration protocol (Figure 2a). The LSV experiment was performed scanning the potential from +1.20 to +0.25 V with a 50 µL drop of solution of 50 mM of the KCl solution. By starting the scan at high potential, oxidation of chloride ions to chlorine gas is produced. Dissolved gas, electrogenerated close to the electrode, is subsequently detected in the reduction scan when the potential sweep reaches lower values. DRP-110CUPH gives rise to a well-defined reduction peak at +0.80 V whereas DRP-110FEPH and DRP-110MNPH electrodes show wider peaks that can be attributed to a stronger interaction with iron and manganese metallic centers. This is consistent with the theoretical studies that predict that the sensitivity depends on the metallic center involved [30]. Although CoPH films have already been employed in the detection of chlorine in previous work [31], no reduction processes are observed using DRP-410 following the methodology developed in this work. Taking into account the better electrochemical response of DRP-110CUPH, further studies have been carried out using these electrodes.

Other chlorinated species have been assayed using DRP-110CUPH electrodes. Depending on the specie, the linear sweep voltammogram profile changes (Figure 2b). When potassium chloride is assayed, only a peak at +0.80 V appears while perchloric acid only shows one peak at around +0.40 V. In addition, hydrochloric acid gives both peaks as is shown in Figure 2b. The different electrochemical behavior of these species observed onto DRP-110CUPH can be understood by taking into account the nature of the copper phthalocyanine. The linear voltammogram profile observed could be explained by two different phenomena: reduction of electrogenerated chlorine onto the copper center at +0.80 V and reduction at +0.40 V of the copper center released when the ring is destructed. The reduction peak observed at around +0.80 V is due to the reduction of ligand complexed to the metallic center as was recently proposed [32]. On the other hand, the reduction peak observed with HCl and HClO_4_ at +0.40 V could be assigned, initially, to the reduction of free copper [33]. This assumption can be confirmed by the existence of this reduction process in HClO_4_ media, since it is also observed when the potential scan starts at lower potentials, like +0.80 V. This effect is due to the following considerations: although perchlorate cannot be oxidized to a higher oxidation state, it is a strong oxidant that could destroy the phthalocyanine ring and oxidize the metallic center. Then, a subsequent electrochemical reduction of free metal could be responsible for this reduction process. This explanation is also consistent with the appearance of this peak in HCl. This specie can be oxidized to chlorine, but can also oxidize copper, so both reduction processes are observed. Destruction of the phthalocyanine ring can be achieved at higher potentials than +1.10 V as has been previously reported [34]. Due to perchloric and hydrochloric acids being very aggressive media, the degradation of the CuPH ring could be possible in these media, leading to the copper center release that is subsequently reduced. The reduction process at +0.40 V is slightly observed when KCl is using at high concentration or in acid media, so the explanation is consistent with experimental results. The oxidation state of copper involved at this potential remains unclear. There are published works, where copper (II) phthalocyanines are employed, that explain the existence of the reduction peak at +0.40 V. The theoretical mechanism in these works involves the release and reduction of the metallic center, but no oxidation state for this redox process is proposed [34]. Other authors observed the existence of a Cu(III) valence state [33] but this assignation is still controverted [32].

**Figure 3:**
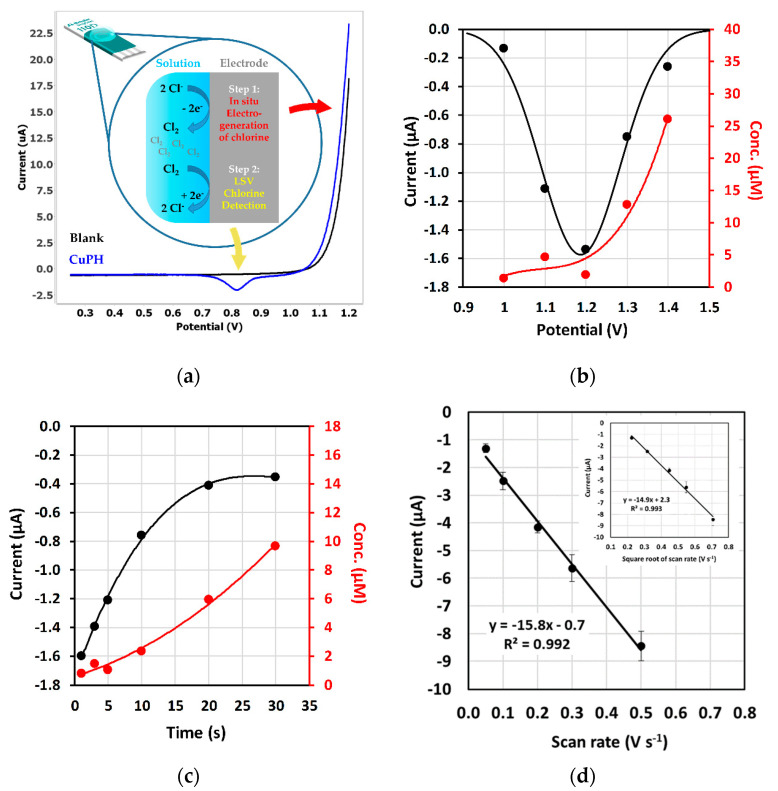
(**a**) Detection scheme and analytical signal obtained with optimized parameters using DRP-110CUPH (blue line) and DRP-110 (black line) in 50 mM KCl solution. The rest of the graphics show optimization of experimental parameters involved in chlorine detection with DRP-110CUPH in 50 mM KCl solution as: (**b**) initial potential, (**c**) time of application of initial potential and (**d**) scan rate (data are given as average ± SD (*n* = 3)).

The analytical signal employed for optimization corresponds to the peak observed at + 0.80 V, where the catalytic reduction of chlorine gas is produced onto the metallic center. The detection scheme developed in this work is shown in Figure 3a, where the two steps involved are explained taking into account the voltammetric profile obtained. Optimum analytical signal is shown in blue color while a blank experiment with a DRP-110 electrode is included in black color for clarification purposes.

Several experimental parameters were optimized to improve the detection purpose. In order to study signal dependency on different experimental conditions vs electrogenerated chlorine, coulometric experiments were also performed. They consist of the application of the same parameter studied in order to collect the charge generated when potential, time or concentration is varied. Assuming a total conversion efficiency of chloride to chlorine in a two-electron oxidation process, it is possible to obtain the chlorine concentration electrogenerated in the 50 µL solution drop employed. Similar experiments were carried out with unmodified electrodes.

Several potentials to generate chlorine were tested from +1.10 to +1.40 V. Optimization of initial potential shows a Gaussian-like dependence of the intensity current of the reduction peak vs. potential, showing the highest value at +1.20 V (Figure 3b). This Gaussian behavior is not consistent with the continuous increase of chlorine concentration with increasing potential, as is shown in the secondary axis in Figure 3b. This could be explained by the destruction of the phthalocyanine ring, in accordance with the suggestions of other authors [34]. Then, + 1.20 V is the optimum initial potential where the amount of chlorine generated from chloride is enough to afford a well-defined reduction peak, while complete destruction of the CuPH ring is avoided.

The time of application of initial potential was also optimized. Several values were assayed from 1 to 30 s. As can be seen in Figure 3c, peak height increases while time decreases. This behavior shows that the analytical signal assigned to the reduction process of electrogenerated chlorine is a time dependent process. In fact, although the concentration of chlorine increases with time, the analytical signal increases with decreasing time. The explanation of this behavior relies on the fact that chlorine has a quick diffusion coefficient [35], so it is necessary to apply short times of initial potential in order to avoid and/or minimize losing the generated chlorine. This result can be confirmed when varying the scan rate. Application of higher scan rates shows an increase of the current peak due to the situation that chlorine gas has no time to diffuse away from electrode surface before its subsequent reduction step. As shown in Figure 3d, there is a linear relationship dependency of peak height with scan rate but also with the square root of scan rate. This means that the electrodic process involved has a hybrid nature, that is, it is controlled by adsorption and diffusion. This is consistent with the fact that chlorine gas should be adsorbed weakly onto the metal center in order to be reduced. Capacitive current also increases with scan rate, so a lower scan rate value is finally selected. Thus, optimal conditions chosen for quantification studies with DRP-110CUPH are an initial potential of + 1.20 V applied during 3 s with a subsequent cathodic linear sweep of potentials with a scan rate of 50 mV s^−1^.

**Figure 4:**
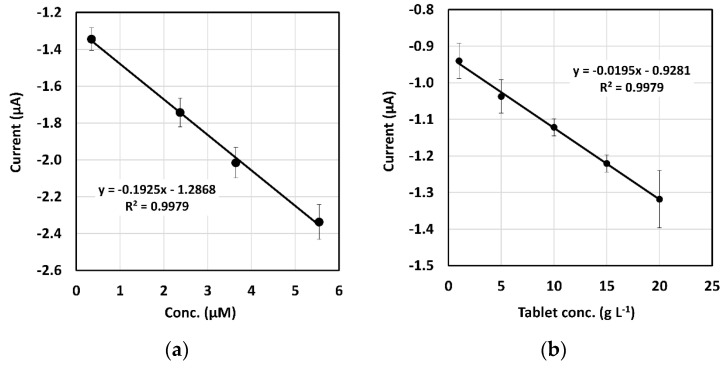
Calibration plots obtained with DRP-110CUPH for (**a**) electrogenerated chlorine and (**b**) chlorine generated with the chlorination tablet.

With the aim to show potential application of these electrodes for the quantification of chlorinated species, a calibration plot with 110CUPH was done. There is a linear range (Figure 4a) with good correlation for concentrations of chlorine comprised from 0.3 to 5.5 µM (i_p_ (µA) = −0.193 [chlorine] µA µM^−1^ − 1.287 µA, R^2^ = 0.998, n = 3).

Taking into account this result, it is possible to make a comparison between existing methodologies for chlorine detection found in the literature and CuPH as a sensing phase (Table 2). Analytical figures of merit are comparable to works involving more complex experimental setup. In this way, the use of screen-printed electrodes offers a new affordable and easy-to-use platform for chlorine sensing.

Due to the sensitivity observed with different concentrations of certain chlorine based species, DRP-110CUPH was tested to quantify these species in real samples. In this way, a proof of concept assay was performed with chlorine tablets typically used to protect reverse osmosis membranes in ultrapure water dispenser systems. The main component of these tablets is sodium dichloroisocyanurate, a common chlorination agent employed as the source of chlorine. It is used in applications where a more user-friendly solution than sodium hypochlorite is required [36]. In a typical experiment, a tablet was crushed in a mortar and different quantities were placed in several tubes. Then, 1 mL ultrapure water was added and mixed by stirring. Once tablet dust is solved, a 50 µL solution drop was placed onto DRP-110CUPH and the LSV method is applied. Optimal experimental conditions were used but the chlorine is chemically generated by tablets. When testing a chlorinated tablet, contrary to what was expected, the reduction peak at +0.80 V was not observed, probably due to matrix effects. However, the reduction process at +0.40 V, related to destruction of the phthalocyanine ring and copper release is visible. This peak was not observed when lower potentials were employed, so its existence can be only assessed to copper release from phthalocyanine as destruction of the ring is probably obtained with the chlorinated tablet. Despite using a different peak for quantification, data show that DRP-110CUPH are also capable to respond quantitatively to different concentration of chlorine tablets. This makes possible a calibration plot (Figure 4b) based on the fact that the more tablet quantity is dissolved, the higher a signal is achieved as more copper is released. A linear relationship is obtained from 1 to 20 g L^−1^ (i_p_ (µA) = −0,02 [tablet] µA L g^−1^ – 0.93 µA, R ^2^ = 0.998, n = 3). This unexpected behavior affords the possibility to use both reduction peaks to detect chlorinated species. Due to their rich electrochemistry, metal phthalocyanines offer interesting voltammetric behavior that can be useful in future sensing applications for chlorinated species detection.

## 4. Conclusions

Four commercial phthalocyanine based SPEs have been characterized by the Raman spectroscopic technique. Although the Raman spectra show that their vibrational bands are located at the same position, the characteristic band between 1510 and 1540 cm^−1^ allows us to quickly identify them because they are metal dependent: 1533 cm^−1^ for CoPH, 1524 cm^−1^ for CuPH, 1512 cm^−1^ for MnPH and 1511 cm^−1^ for FePH. Furthermore, a reversible up-shifting of these fingerprint bands is detected when the electrode is wet. This effect suggests the modification of the solvation sphere due to the introduction of solvent molecules in the structure.

Chloride based species such as KCl and HCl were tested with the metal phthalocyanine modified electrodes as DRP-410, DRP-110CUPH, DRP-110FEPH and DRP-110MNPH. Sensors assayed show different voltammetric profiles depending on the reagent and the metal phthalocyanine employed. DRP-110CUPH electrochemical behavior was further discussed taking into account two electrodic processes observed on the voltammograms. One of them is related to the reduction of chlorine onto the metal center and the other one is associated with the reduction of the copper releases from the phthalocyanine complex. Promising results were obtained with DRP-110CUPH, so an optimization step was done to clarify parameter dependence with the detection scheme developed. A two step electrochemical assay was performed. Firstly, chlorine gas is electrogenerated applying an initial potential of +1.20 V during 3 s. Secondly, an LSV scan from +1.20 to +0.25 V at a rate of 50 mV s^−1^ detects chlorine diluted in solution. Optimized procedures were applied to quantify chlorine generated when oxidizing potassium chloride, obtaining good correlation. Finally, the method developed in this work was successfully applied with a purifying commercial tablet as chlorine precursor. The proof of concept assay presented can serve as a starting point for future sensing applications of these novel sensing phases.

## Figures and Tables

**Table 1:** Assignment of Raman bands of dry and wet CoPH (DRP-410), CuPH (DRP-110CUPH), MnPH (DRP-110MNPH) and FePH (DRP-110FEPH) [28].

DRP-410 Raman Bands (cm^−1^)		DRP-110CUPH Raman Bands (cm^−1^)		DRP-110MNPH Raman Bands (cm^−1^)		DRP-110FEPH Raman Bands (cm^−1^)		Assignment
dry	wet	dry	wet	dry	wet	dry	wet	
480	483	480	483	483	486	480	483	C_β_-C_γ_-C_δ_, C_δ_-C_δ_-H, C_α_-C_β_-C_γ_
593	593	590	590	590	593	587	587	ring breathing
680	683	677	680	674	674	677	677	C_α_-N_β_-C_α_, N_α_-C_α_-C_β_, C_β_-C_γ_-C_δ_
745	748	745	745	745	748	745	745	C_α_-N_α_-C_α_, N_α_-C_α_-N_β_, C_γ_-C_δ_-C_δ_, C_α_-N_β_-C_α_
779	779	772	776	779	779	772	772	C_β_-C_γ_-H, C_γ_-C_δ_-H out-of-plane
829	833	829	829	829	829	826	826	C_α_-N_α_-C_α_, C_α_-N_β_-C_α_, N_α_-C_α_-N_β_
954	957	951	951	944	944	944	944	C_β_-C_γ_-H, C_γ_-C_δ_-H, C_δ_-C_δ_-H
1005	1006	1002	1006	1004	1004	1002	1005	C_β_-C_γ_-H, C_γ_-C_δ_-C_δ_, C_δ_-C_δ_-H
1107	1107	1107	1107	1104	1100	1104	1107	C_β_-C_γ_-H, C_δ_-C_δ_-H, C_α_-N_α_
1138	1138	1141	1141	1144	1144	1138	1138	C_β_-C_γ_-H, C_δ_-C_δ_-H, C_α_-N_α_-C_α_
1184	1187	1191	1194	1184	1184	1181	1181	C_α_-N_α_-C_α_, isoindole breathing, C_δ_-C_δ_-H, C_δ_-C_γ_-H
1212	1212	1215	1212	1206	1206	1212	1212	C_α_-N_α_-C_α_, isoindole deformation, C_β_-C_γ_-H, C_δ_-C_δ_-H
1306	1306	1306	1306	1306	1308	1303	1308	C_β_-C_γ_-H, C_γ_-C_δ_-H, C_α_-C_β_-C_β_
1335	1338	1338	1341	1335	1338	1332	1338	ring deformation
1452	1455	1446	1449	1446	1449	1446	1449	C_γ_-C_δ_-H, C_δ_-C_δ_-H, C_β_-C_γ_
1533	1541	1524	1528	1512	1518	1511	1523	C_α_-N_β_-C_α_

**Table 2:** Comparison chart of the analytical figure of merit among this work and the state of the art found in literature with works employing copper phthalocyanines as the sensing phase for chlorine detection.

Sensing Phase	Parameter Measured	Linear Range (ppb)	LOD (ppb) *	Ref.
Thin film of Hexadecafluorinated CuPh with carbon nanotubes	Resistance	0 to 2000 Curve fitting	0.27	[23]
CuPH thin film	Conductivity	180 to 35,000	180	[25]
Sulphonated CuPH film	Conductance	5 to 2000	5	[26]
SPE based CuPH	Peak current	21 to 390	9.7	This work

* LOD: Limit of detection.
